# Diamminecopper(I) divanadate(IV,V)

**DOI:** 10.1107/S2414314626008862

**Published:** 2026-08-28

**Authors:** Hibiki Kunisawa, Ryutaro Okuma, Toshihiro Nomura

**Affiliations:** a Department of Physics, Shizuoka University, Shizuoka 422-8529, Japan; b Institute for Solid State Physics, The University of Tokyo, Kashiwa, Chiba, 277-8581, Japan; Vienna University of Technology, Austria

**Keywords:** crystal structure, vanadium, mixed valence, copper(I) ammine, layered structure

## Abstract

The crystal structure of diamminecopper(I) divanadate(IV,V), or poly[di­amminecopper(I) [tri-μ_3_-oxido-di-μ_2_-oxido-divanadium(IV,V)]], {[Cu(NH_3_)_2_]V_2_O_5_}_*n*_, has been determined by single-crystal X-ray diffraction. The compound crystallizes in the monoclinic space group *P*2_1_/*c* and consists of infinite {V_2_O_5_}^−^ layers expanding parallel to the *bc* plane built from edge- and corner-sharing [VO_5_] square pyramids. Almost linear diamminecopper(I) cations, [Cu(NH_3_)_2_]^+^, are located between the vanadate layers and are linked to the framework through N—H⋯O hydrogen bonds.

## Abstract 2

[Cu(NH_3_)_2_]V_2_O_5_ contains layers of {V_2_O_5_}^−^ anions expanding in the *bc* plane, separated by almost linear [Cu(NH_3_)_2_]^+^ cations.

## Graphical abstract

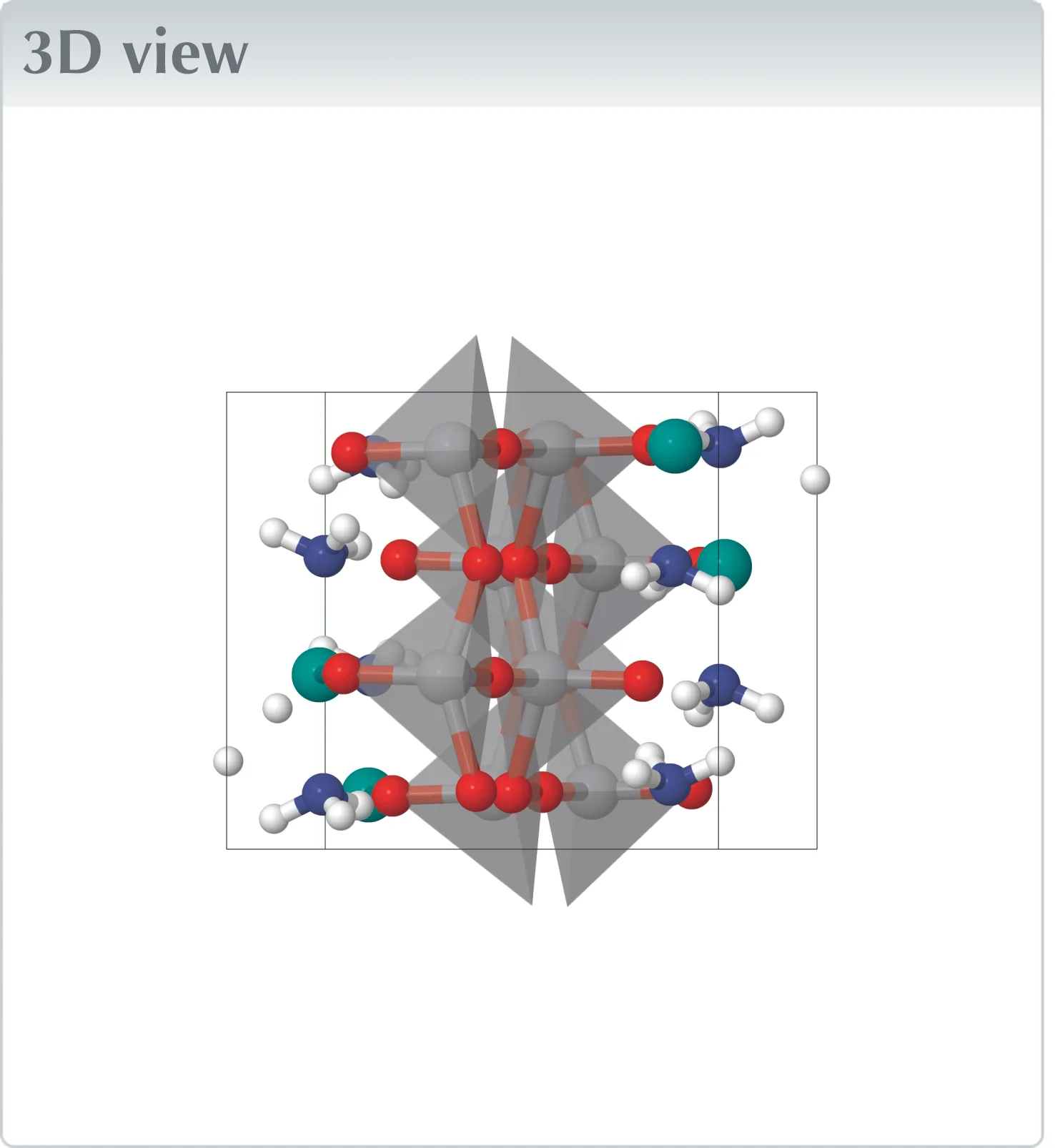

## Structure description

Layered vanadates constitute an extensive family of compounds (Zavalij & Whittingham, 1999 ▸; Chernova *et al.*, 2009 ▸; Hu *et al.*, 2023 ▸). Among them, *α*’-NaV_2_O_5_ (Meetsma *et al.*, 1998 ▸) and CaV_2_O_5_ (Onoda & Nishiguchi, 1996 ▸) are representative phases containing layers of corner- and edge-sharing [VO_5_] square pyramids. Herein, we report the crystal structure of the new layered vanadate, [Cu(NH_3_)_2_]V_2_O_5_.

**Figure 1:**
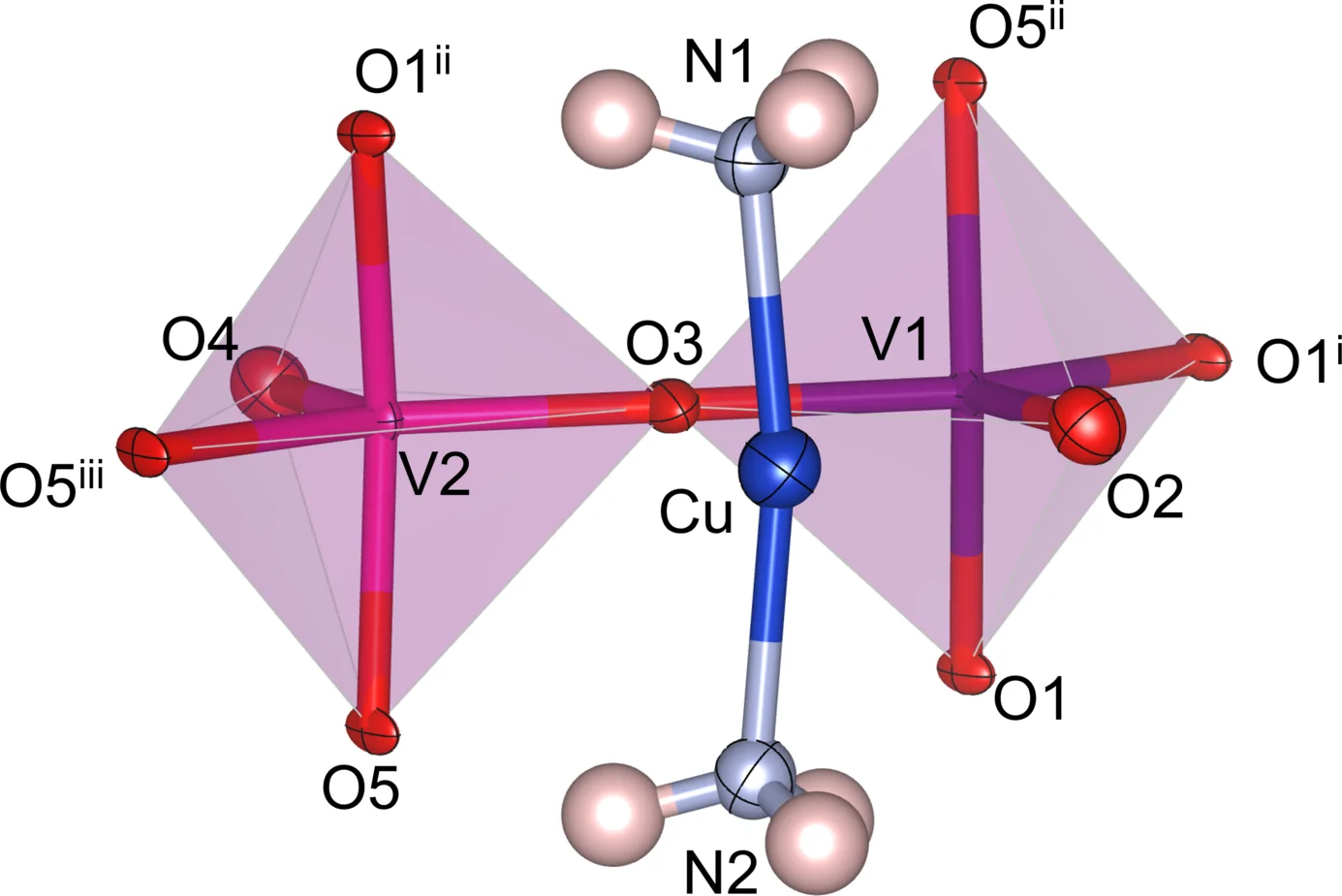
The asymmetric unit of the title compound expanded to show the complete [VO_5_] square-pyramidal units. Displacement ellipsoids are drawn at the 50% probability level; symmetry codes refer to Table 1 ▸.

**Figure 3:**
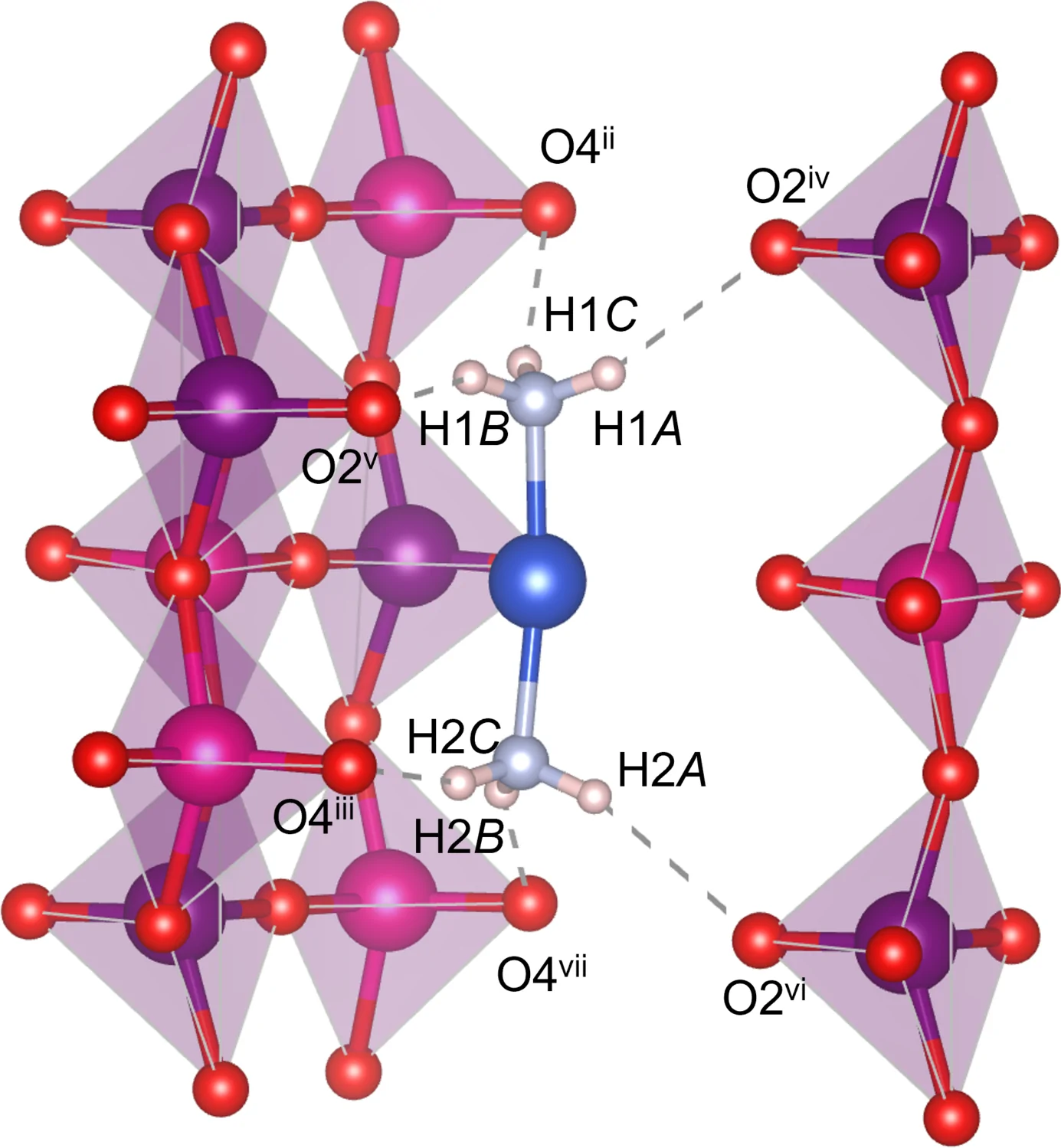
Hydrogen-bonding network in the title compound. The relatively weak bonds (N1—H1*B*⋯O1^ii^ and N2—H2*C*⋯O5) are omitted for clarity; symmetry codes refer to Table 2 ▸.

The structure of the title compound consists of infinite {V_2_O_5_}^−^ layers expanding parallel to the *bc* plane separated by complex [Cu(NH_3_)_2_]^+^ cations (Figs. 1 ▸ and 2 ▸, Table 1 ▸). Both V1 and V2 atoms are coordinated by five oxygen atoms in slightly distorted square-pyramidal shapes, with *τ*_5_ values of 0.11 (V1) and 0.03 (V2), respectively (the *τ*_5_ value for an ideal square pyramid is 0, and for an ideal trigonal bipyramid is 1; Addison *et al.*, 1984 ▸). The nearest-neighbor [VO_5_] square pyramids, with apical O2 and O4 atoms pointing alternately upward (U) and downward (D) along the *a* axis are edge-shared *via* the μ_3_-O1 and μ_3_-O5 atoms to form zigzag chains running parallel to the *b* axis (represented as {UD} chains in Zavalij’s notation (Zavalij & Whittingham, 1999 ▸), which is used hereafter). The {UD} chains are linked by corner-sharing *via* the μ_2_-O3 atoms along the *c* axis, giving rise to ({UD}.{DU}.) layers. The almost linear [Cu(NH_3_)_2_]^+^ complexes [the N1—Cu—N2 angle is 170.5 (2)°] are aligned parallel to the the *b* axis. The two ammine ligands adopt an eclipsed conformation relative to the N—Cu—N backbone, which is consolidated by N—H⋯O hydrogen bonds to the anionic layers (Fig. 3 ▸, Table 2 ▸). We note that H1*B* and H2*C* participate in bifurcated hydrogen bonds, although the weaker ones are omitted in Fig. 3 ▸.

Bond-valence-sum (BVS) calculations (Brown, 2002 ▸; Brese & O’Keeffe, 1991 ▸) were performed to estimate the oxidation states of Cu, V1, and V2 cations (Table 3 ▸). The BVS values (in valence units) of Cu, V1, and V2 are 1.03, 4.56–4.80, and 4.41–4.65, respectively, depending on the parameters adopted for V^IV^ and V^V^. These values support that the oxidation state of Cu is +1, while V1 and V2 are in mixed-valence states between +4 and +5, giving an average oxidation state of +4.5 for vanadium. We comment that the precise assignment of charges in such mixed-valence vanadates can be challenging because the V—O bond lengths largely depend on the coordination number and the function (bridging or terminal) of the coordinating oxygen atoms (Weil *et al.*, 2007 ▸), although the BVS values reasonably support the expected oxidation states. We note that the temperature dependence of the electrical conductivity of the title compound exhibits semiconducting behavior.

According to the Inorganic Crystal Structure Database (ICSD; version 2025–1; Zagorac *et al.*, 2019 ▸), [Cu(NH_3_)_2_]V_2_O_5_ is the first layered vanadate containing amminecopper(I) cations. In contrast, [Cu(NH_3_)_2_](VO_3_)_2_ (Chrappová *et al.*, 2008 ▸) and {VO(O_2_)_2_(NH_3_)}_2_{μ-Cu(NH_3_)_4_} (Aschwanden *et al.*, 1993 ▸) comprise copper and vanadium atoms in oxidation states of +2 and +5, respectively, and do not have infinite vanadate layers. Here, we discuss the crystal structure of the title compound in relation to *α*′-NaV_2_O_5_ which has a similar mixed-valence state and layered network of [VO_5_] square pyramids. First, [Cu(NH_3_)_2_]V_2_O_5_ has a monoclinic structure (space group *P*2_1_/*c*), while *α*-NaV_2_O_5_ crystallizes in an ortho­rhom­bic structure (space group *Pmmn*). Second, {UD} chains of [VO_5_] square pyramids form ({UD}.{DU}.) layers in [Cu(NH_3_)_2_]V_2_O_5_, while they form ({UD}.{UD}.) layers in *α*′-NaV_2_O_5_. These differences may be related to the larger size and slightly bent shape of the complex [Cu(NH_3_)_2_]^+^ cation. Consequently, the separation between adjacent vanadate layers is approximately 7.75 Å in [Cu(NH_3_)_2_]V_2_O_5_, which is considerably larger than the 4.80 Å in *α*′-NaV_2_O_5_.

## Synthesis and crystallization

Single crystals of [Cu(NH_3_)_2_]V_2_O_5_ were obtained by electrochemical-hydro­thermal synthesis using a custom-built PTFE-lined autoclave equipped with Cu electrodes. Basic copper carbonate (0.25 g), V_2_O_5_ (0.3 g), and 5 ml of 5%_wt_ aqueous ammonia were placed in the autoclave and heated at 423 K for 24 h while applying 3 V. All reagents were purchased from FUJIFILM Wako and used without further purification. Black, plate-like crystals were grown on the cathode, and a single crystal was selected for X-ray diffraction measurement at room temperature. We note that the crystals might gradually degrade in air, and even under vacuum, on a timescale of a few days.

## Refinement

The crystallographic data, data collection and structure refinement are summarized in Table 4 ▸. All ammine hydrogen atoms were located in difference-Fourier maps and refined using the *DFIX* and *DANG* restraints (Sheldrick, 2015*b* ▸).

## Supplementary Material

Crystal structure: contains datablock(s) I. DOI: 10.1107/S2414314626008862/wm4258sup1.cif

Structure factors: contains datablock(s) I. DOI: 10.1107/S2414314626008862/wm4258Isup2.hkl

CCDC reference: 2583502

Additional supporting information:  crystallographic information; 3D view; checkCIF report

## Figures and Tables

**Figure 2:**
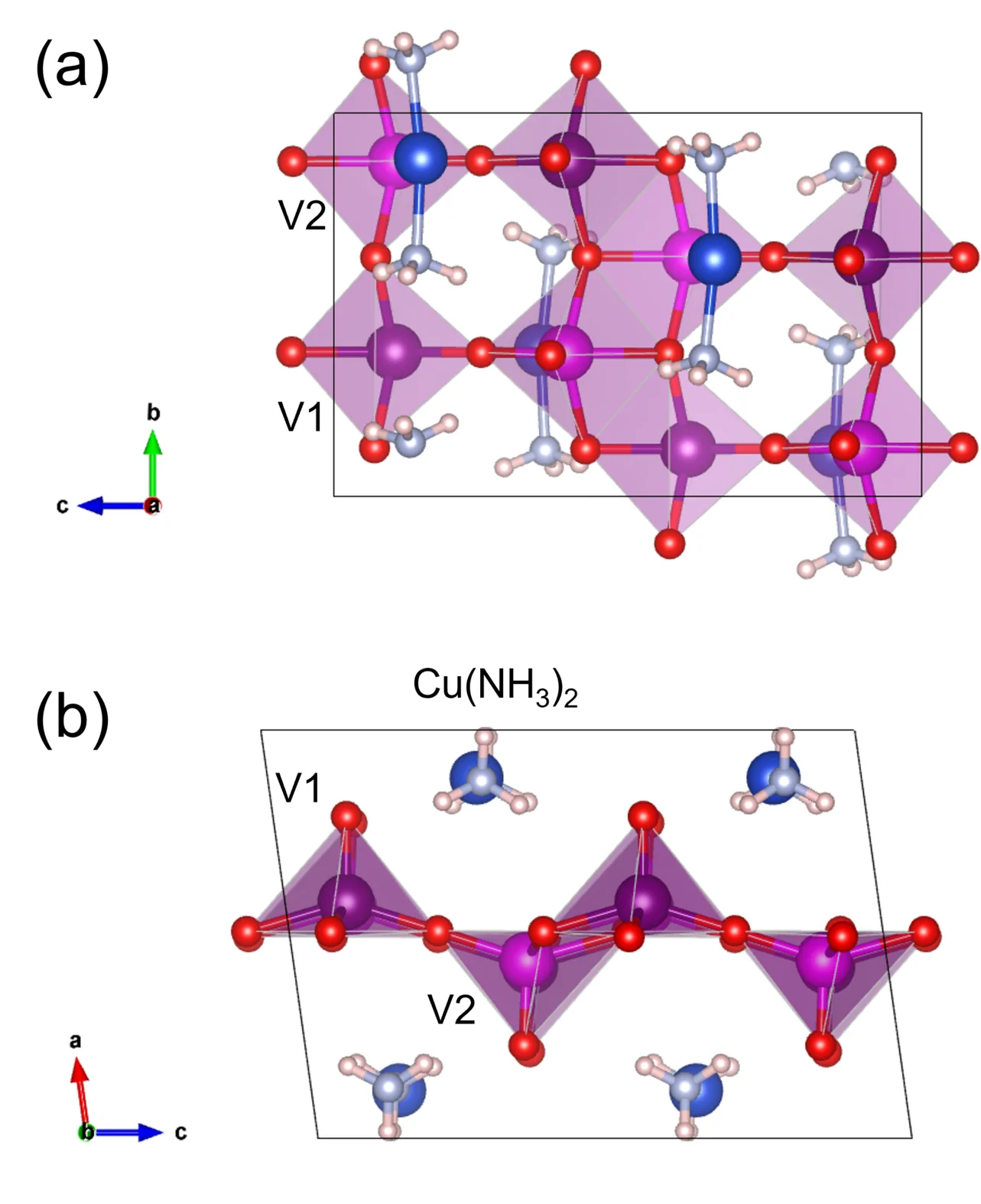
The unit cell of the title compound with polyhedral representation viewed along (*a*) the *a* and (*b*) the *b* axes. V1 and V2 are shown by dark and light purple spheres, respectively.

**Table 1:** Selected bond lengths (Å)

V1—O1^i^	1.964 (3)	V2—O3	1.779 (2)
V1—O1	1.9275 (19)	V2—O4	1.615 (3)
V1—O2	1.622 (3)	V2—O5	1.9208 (18)
V1—O3	1.829 (2)	V2—O5^iii^	1.974 (3)
V1—O5^ii^	1.9258 (18)	Cu—N1	1.906 (3)
V2—O1^ii^	1.9253 (19)	Cu—N2	1.897 (4)

Symmetry codes: (i) 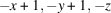; (ii) 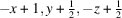; (iii) 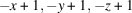.

**Table 2:** Hydrogen-bond geometry (Å, °)

*D*—H⋯*A*	*D*—H	H⋯*A*	*D*⋯*A*	*D*—H⋯*A*
N1—H1*A*⋯O2^iv^	0.90 (2)	2.25 (2)	3.124 (5)	164 (4)
N1—H1*B*⋯O1^ii^	0.89 (2)	2.44 (3)	3.096 (5)	131 (3)
N1—H1*B*⋯O2^v^	0.89 (2)	2.36 (3)	3.147 (5)	148 (4)
N1—H1*C*⋯O4^ii^	0.88 (2)	2.33 (2)	3.168 (4)	159 (4)
N2—H2*A*⋯O2^vi^	0.90 (2)	2.30 (2)	3.167 (5)	163 (4)
N2—H2*B*⋯O4^vii^	0.89 (2)	2.30 (2)	3.158 (5)	162 (4)
N2—H2*C*⋯O4^iii^	0.88 (2)	2.35 (2)	3.163 (5)	154 (4)
N2—H2*C*⋯O5	0.88 (2)	2.57 (4)	3.155 (5)	124 (3)

Symmetry codes: (ii) 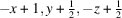; (iii) 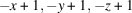; (iv) 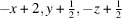; (v) 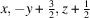; (vi) 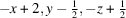; (vii) 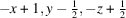.

**Table 3:** Bond-valence sum calculation (in valence units) using the parameters of V^IV^ (left) and V^V^ (right)

Cu	1.03	1.03
V1	4.56	4.80
V2	4.41	4.65
O1	1.98	2.08
O2	1.65	1.73
O3	1.90	2.00
O4	1.63	1.71
O5	1.97	2.07

**Table 4:** Experimental details

Crystal data	
Chemical formula	[Cu(NH_3_)_2_]V_2_O_5_
*M*_r_	279.50
Crystal system, space group	Monoclinic, *P*2_1_/*c*
Temperature (K)	293
*a*, *b*, *c* (Å)	7.8226 (3), 7.2686 (3), 11.2634 (4)
β (°)	97.994 (4)
*V* (Å^3^)	634.21 (4)
*Z*	4
Radiation type	Cu *K*α
μ (mm^−1^)	27.97
Crystal size (mm)	0.100 × 0.05 × 0.02

Data collection	
Diffractometer	XtaLAB Synergy R, HyPix
Absorption correction	Multi-scan (*CrysAlis PRO*; Rigaku OD, 2023 ▸)
*T*_min_, *T*_max_	0.464, 1.000
No. of measured, independent and observed [*I* > 2σ(*I*)] reflections	3595, 1268, 1010
*R*_int_	0.034
(sin θ/λ)_max_ (Å^−1^)	0.630

Refinement	
*R*[*F*^2^ > 2σ(*F*^2^)], *wR*(*F*^2^), *S*	0.045, 0.126, 0.99
No. of reflections	1268
No. of parameters	109
No. of restraints	12
H-atom treatment	Only H-atom coordinates refined
Δρ_max_, Δρ_min_ (e Å^−3^)	1.11, −0.97

Computer programs: *CrysAlis PRO* (Rigaku OD, 2023 ▸), *SHELXT* (Sheldrick, 2015*a* ▸), *SHELXL* (Sheldrick, 2015*b* ▸), *VESTA* (Momma & Izumi, 2011 ▸) and *OLEX2* (Dolomanov *et al.*, 2009 ▸).

## full crystallographic data

### Poly[diamminecopper(I) [tri-µ3-oxido-di-µ2-oxido-divanadium(IV,V)]] Crystal data

**Table:**

[Cu(NH_3_)_2_]V_2_O_5_	*F*(000) = 540
*M**_r_* = 279.50	*D*_x_ = 2.927 Mg m^−^^3^
Monoclinic, *P*2_1_/*c*	Cu *K*α radiation, λ = 1.54184 Å
*a* = 7.8226 (3) Å	Cell parameters from 1772 reflections
*b* = 7.2686 (3) Å	θ = 5.7–75.7°
*c* = 11.2634 (4) Å	µ = 27.97 mm^−^^1^
β = 97.994 (4)°	*T* = 293 K
*V* = 634.21 (4) Å^3^	Block, black
*Z* = 4	0.1 × 0.05 × 0.02 mm

### Poly[diamminecopper(I) [tri-µ3-oxido-di-µ2-oxido-divanadium(IV,V)]] Data collection

**Table:**

XtaLAB Synergy R, HyPix diffractometer	1010 reflections with *I* > 2σ(*I*)
Detector resolution: 10.0000 pixels mm^-1^	*R*_int_ = 0.034
ω scans	θ_max_ = 76.2°, θ_min_ = 5.7°
Absorption correction: multi-scan (CrysAlisPro; Rigaku OD, 2023)	*h* = −9→9
*T*_min_ = 0.464, *T*_max_ = 1.000	*k* = −8→8
3595 measured reflections	*l* = −14→14
1268 independent reflections	

### Poly[diamminecopper(I) [tri-µ3-oxido-di-µ2-oxido-divanadium(IV,V)]] Refinement

**Table:**

Refinement on *F*^2^	12 restraints
Least-squares matrix: full	Hydrogen site location: difference Fourier map
*R*[*F*^2^ > 2σ(*F*^2^)] = 0.045	Only H-atom coordinates refined
*wR*(*F*^2^) = 0.126	*w* = 1/[σ^2^(*F*_o_^2^) + (0.0948*P*)^2^] where *P* = (*F*_o_^2^ + 2*F*_c_^2^)/3
*S* = 0.99	(Δ/σ)_max_ < 0.001
1268 reflections	Δρ_max_ = 1.11 e Å^−^^3^
109 parameters	Δρ_min_ = −0.97 e Å^−^^3^

### Poly[diamminecopper(I) [tri-µ3-oxido-di-µ2-oxido-divanadium(IV,V)]] Special details

**Table:**

Geometry. All esds (except the esd in the dihedral angle between two l.s. planes) are estimated using the full covariance matrix. The cell esds are taken into account individually in the estimation of esds in distances, angles and torsion angles; correlations between esds in cell parameters are only used when they are defined by crystal symmetry. An approximate (isotropic) treatment of cell esds is used for estimating esds involving l.s. planes.

### Poly[diamminecopper(I) [tri-µ3-oxido-di-µ2-oxido-divanadium(IV,V)]] Fractional atomic coordinates and isotropic or equivalent isotropic displacement parameters (Å^2^)

**Table:**

	*x*	*y*	*z*	*U*_iso_*/*U*_eq_	
V1	0.57848 (8)	0.62325 (6)	0.10265 (5)	0.0106 (3)	
V2	0.43650 (8)	0.62603 (6)	0.39401 (5)	0.0109 (3)	
Cu	0.88241 (9)	0.61843 (7)	0.35201 (5)	0.0323 (3)	
O1	0.4936 (4)	0.3765 (2)	0.0715 (2)	0.0139 (6)	
O2	0.7877 (4)	0.6161 (3)	0.1236 (3)	0.0235 (7)	
O3	0.5081 (3)	0.6245 (2)	0.25083 (18)	0.0221 (8)	
O4	0.2282 (4)	0.6324 (3)	0.3686 (3)	0.0235 (7)	
O5	0.4937 (4)	0.3735 (2)	0.4307 (2)	0.0140 (6)	
N1	0.8759 (5)	0.8803 (4)	0.3594 (4)	0.0258 (9)	
H1A	0.979 (3)	0.934 (6)	0.378 (4)	0.039*	
H1B	0.815 (5)	0.905 (6)	0.418 (3)	0.039*	
H1C	0.826 (5)	0.927 (6)	0.291 (2)	0.039*	
N2	0.8750 (6)	0.3591 (5)	0.3697 (4)	0.0281 (9)	
H2A	0.980 (3)	0.308 (7)	0.384 (4)	0.042*	
H2B	0.821 (5)	0.299 (6)	0.306 (3)	0.042*	
H2C	0.821 (5)	0.336 (7)	0.432 (3)	0.042*	

### Poly[diamminecopper(I) [tri-µ3-oxido-di-µ2-oxido-divanadium(IV,V)]] Atomic displacement parameters (Å^2^)

**Table:**

	*U*^11^	*U*^22^	*U*^33^	*U*^12^	*U*^13^	*U*^23^
V1	0.0167 (4)	0.0089 (4)	0.0065 (4)	−0.00041 (18)	0.0025 (3)	0.00007 (16)
V2	0.0176 (4)	0.0097 (4)	0.0058 (4)	0.00023 (18)	0.0028 (3)	0.00032 (16)
Cu	0.0422 (5)	0.0271 (4)	0.0292 (4)	−0.0001 (2)	0.0102 (3)	−0.0014 (2)
O1	0.0278 (15)	0.0084 (12)	0.0049 (12)	−0.0010 (8)	0.0000 (10)	0.0001 (7)
O2	0.0238 (15)	0.0243 (15)	0.0221 (15)	−0.0010 (9)	0.0018 (11)	0.0007 (9)
O3	0.042 (2)	0.0112 (15)	0.0154 (17)	−0.0009 (9)	0.0122 (15)	−0.0006 (7)
O4	0.0229 (16)	0.0248 (15)	0.0224 (15)	−0.0002 (9)	0.0012 (11)	0.0015 (9)
O5	0.0297 (16)	0.0092 (12)	0.0026 (12)	−0.0013 (8)	0.0007 (11)	0.0002 (7)
N1	0.025 (2)	0.025 (2)	0.029 (2)	0.0000 (13)	0.0082 (17)	0.0001 (12)
N2	0.028 (2)	0.0270 (18)	0.030 (2)	0.0001 (14)	0.0053 (16)	−0.0012 (13)

### Poly[diamminecopper(I) [tri-µ3-oxido-di-µ2-oxido-divanadium(IV,V)]] Geometric parameters (Å, º)

**Table:**

V1—V1^i^	3.0455 (11)	V2—O5	1.9208 (18)
V1—V2^ii^	3.0579 (8)	V2—O5^iv^	1.974 (3)
V1—O1^i^	1.964 (3)	Cu—N1	1.906 (3)
V1—O1	1.9275 (19)	Cu—N2	1.897 (4)
V1—O2	1.622 (3)	N1—H1A	0.896 (18)
V1—O3	1.829 (2)	N1—H1B	0.885 (18)
V1—O5^iii^	1.9258 (18)	N1—H1C	0.879 (18)
V2—V2^iv^	3.0634 (10)	N2—H2A	0.897 (18)
V2—O1^iii^	1.9253 (19)	N2—H2B	0.892 (18)
V2—O3	1.779 (2)	N2—H2C	0.882 (18)
V2—O4	1.615 (3)		
			
V1^i^—V1—V2^ii^	72.67 (2)	O4—V2—V2^iv^	111.39 (11)
O1^i^—V1—V1^i^	38.07 (5)	O4—V2—O1^iii^	105.08 (12)
O1—V1—V1^i^	38.92 (7)	O4—V2—O3	106.08 (15)
O1^i^—V1—V2^ii^	37.72 (5)	O4—V2—O5	105.32 (12)
O1—V1—V2^ii^	109.59 (7)	O4—V2—O5^iv^	107.99 (14)
O1—V1—O1^i^	76.99 (10)	O5—V2—V1^v^	110.82 (7)
O2—V1—V1^i^	112.58 (10)	O5^iv^—V2—V1^v^	37.80 (5)
O2—V1—V2^ii^	112.47 (10)	O5^iv^—V2—V2^iv^	37.52 (5)
O2—V1—O1	108.19 (11)	O5—V2—V2^iv^	38.75 (7)
O2—V1—O1^i^	106.81 (14)	O5—V2—O1^iii^	144.03 (13)
O2—V1—O3	107.03 (14)	O5—V2—O5^iv^	76.28 (9)
O2—V1—O5^iii^	108.72 (12)	N2—Cu—N1	170.5 (2)
O3—V1—V1^i^	123.72 (7)	V1—O1—V1^i^	103.01 (10)
O3—V1—V2^ii^	125.12 (7)	V2^vi^—O1—V1^i^	103.68 (10)
O3—V1—O1^i^	146.15 (13)	V2^vi^—O1—V1	139.65 (15)
O3—V1—O1	91.78 (10)	V2—O3—V1	179.16 (16)
O3—V1—O5^iii^	93.22 (9)	V1^vi^—O5—V2^iv^	103.28 (9)
O5^iii^—V1—V1^i^	109.34 (7)	V2—O5—V1^vi^	143.85 (15)
O5^iii^—V1—V2^ii^	38.92 (7)	V2—O5—V2^iv^	103.72 (9)
O5^iii^—V1—O1^i^	76.63 (9)	Cu—N1—H1A	114 (3)
O5^iii^—V1—O1	139.36 (13)	Cu—N1—H1B	105 (3)
V1^v^—V2—V2^iv^	73.36 (2)	Cu—N1—H1C	111 (3)
O1^iii^—V2—V1^v^	38.61 (7)	H1A—N1—H1B	108 (3)
O1^iii^—V2—V2^iv^	110.85 (7)	H1A—N1—H1C	108 (3)
O1^iii^—V2—O5^iv^	76.41 (9)	H1B—N1—H1C	110 (3)
O3—V2—V1^v^	125.01 (7)	Cu—N2—H2A	113 (3)
O3—V2—V2^iv^	126.42 (8)	Cu—N2—H2B	115 (3)
O3—V2—O1^iii^	94.16 (10)	Cu—N2—H2C	107 (3)
O3—V2—O5^iv^	145.92 (12)	H2A—N2—H2B	105 (3)
O3—V2—O5	95.49 (9)	H2A—N2—H2C	108 (3)
O4—V2—V1^v^	111.63 (11)	H2B—N2—H2C	108 (3)

Symmetry codes: (i) −*x*+1, −*y*+1, −*z*; (ii) *x*, −*y*+3/2, *z*−1/2; (iii) −*x*+1, *y*+1/2, −*z*+1/2; (iv) −*x*+1, −*y*+1, −*z*+1; (v) *x*, −*y*+3/2, *z*+1/2; (vi) −*x*+1, *y*−1/2, −*z*+1/2.

### Poly[diamminecopper(I) [tri-µ3-oxido-di-µ2-oxido-divanadium(IV,V)]] Hydrogen-bond geometry (Å, º)

**Table:**

*D*—H···*A*	*D*—H	H···*A*	*D*···*A*	*D*—H···*A*
N1—H1*A*···O2^vii^	0.90 (2)	2.25 (2)	3.124 (5)	164 (4)
N1—H1*B*···O1^iii^	0.89 (2)	2.44 (3)	3.096 (5)	131 (3)
N1—H1*B*···O2^v^	0.89 (2)	2.36 (3)	3.147 (5)	148 (4)
N1—H1*C*···O4^iii^	0.88 (2)	2.33 (2)	3.168 (4)	159 (4)
N2—H2*A*···O2^viii^	0.90 (2)	2.30 (2)	3.167 (5)	163 (4)
N2—H2*B*···O4^vi^	0.89 (2)	2.30 (2)	3.158 (5)	162 (4)
N2—H2*C*···O4^iv^	0.88 (2)	2.35 (2)	3.163 (5)	154 (4)
N2—H2*C*···O5	0.88 (2)	2.57 (4)	3.155 (5)	124 (3)

Symmetry codes: (iii) −*x*+1, *y*+1/2, −*z*+1/2; (iv) −*x*+1, −*y*+1, −*z*+1; (v) *x*, −*y*+3/2, *z*+1/2; (vi) −*x*+1, *y*−1/2, −*z*+1/2; (vii) −*x*+2, *y*+1/2, −*z*+1/2; (viii) −*x*+2, *y*−1/2, −*z*+1/2.
